# Associations of dietary phytosterols with blood lipid profiles and prevalence of obesity in Chinese adults, a cross-sectional study

**DOI:** 10.1186/s12944-018-0703-y

**Published:** 2018-03-16

**Authors:** Yan-chuan Li, Chun-long Li, Rui Li, Yang Chen, Meng Zhang, Pan-pan Guo, Dan Shi, Xiao-ning Ji, Ren-nan Feng, Chang-hao Sun

**Affiliations:** 1 Department of Nutrition and Food Hygiene, School of Public Health, Harbin Medical University, Harbin, People’s Republic of China; 2 Department of General Surgery, the Second Affiliated Hospital of Harbin Medical University, Harbin, People’s Republic of China; 3 Department of Food and School Hygiene, Dalian Center for Disease Control and Prevention, Dalian, People’s Republic of China

**Keywords:** Human, dietary phytosterols, TC, LDLc, Obesity

## Abstract

**Background:**

It has been established in RCTs that high dose of phytosterols can significantly reduce blood cholesterol. However, it was uncertain whether low dose of phytosterols from daily diets was effective. In this study, we evaluated the associations between dietary phytosterols and body mass index (BMI), waist circumference (WC), blood glucose, serum lipid profiles and prevalence of overweight/obesity and abdominal obesity in healthy subjects.

**Methods:**

Four hundred nine men and 503 women aged 18–60 years were included in this study. Dietary intakes of phytosterols were estimated using a validated food frequency questionnaire. Height, body weight, WC and blood pressure were measured, an oral glucose tolerance test was performed. Moreover, fasting serum triglyceride (TG), total cholesterol (TC), high density lipoprotein cholesterol (HDLc) and low density lipoprotein cholesterol (LDLc) were further determined.

**Results:**

When comparing extreme quartiles of dietary phytosterols, significant differences of BMI, WC, systolic blood pressure (SBP), diastolic blood pressure (DBP), serum TC and LDLc were found. Dietary phytosterols presented a negative association with BMI, WC, SBP, DBP, serum TC and LDLc (with and without adjustment for energy). After adjustment for confounders, we found higher dietary phytosterols were linked with lower prevalence of overweight/obesity (OR highest vs. lowest quartile = 0.487; 95% CI 0.234, 0.918 for men; OR highest vs. lowest quartile = 0.277; 95% CI 0.124, 0.619 for women) and abdominal obesity (OR highest vs. lowest quartile = 0.344; 95% CI 0.144, 0.819 for men; OR highest vs. lowest quartile = 0.321; 95% CI 0.140, 0.571 for women).

**Conclusions:**

Higher dietary phytosterols were associated with lower BMI, WC, blood pressure, serum TC and LDLc and lower prevalence of overweight/obesity and abdominal obesity in Chinese adults.

**Electronic supplementary material:**

The online version of this article (10.1186/s12944-018-0703-y) contains supplementary material, which is available to authorized users.

## Background

Obesity is an increasing challenge for public health in many countries [1], and although elevated body mass index (BMI) is a modifiable risk factor for a series of metabolic disorders and chronic diseases, including insulin resistance [2], metabolic syndrome, type 2 diabetes [3], cardiovascular diseases (CVDs) [2], hypertension [4], dyslipidemia [5], and certain cancers [6], it is difficult to maintain weight loss. Dietary habits were considered to play a vital role in development, treatment and prevention of chronic diseases, such as obesity, hyperlipidemia, diabetes and CVDs [7–9]. Therefore, it is important to extensively focus on the associations between daily diet and chronic diseases, and reasonable dietary recommendation should be raised.

Plant sterols and plant stanols (together they are referred as phytosterols) are a group of non-nutritive but bioactive compounds naturally present in all plant origin foods, they are 28- or 29-carbon alcohols and their structures are similar to cholesterol, a 27-carbon alcohol [10]. The dominant dietary plant sterols are β-sitosterol, campesterol, and stigmasterol, all have double bonds at the C-5 position of the ring. In certain structures, the double bond is saturated and these compounds are referred as plant stanols. Compared with plant sterols, concentrations of plant stanols, such as β-sitostanol and campestanol were generally lower in daily foods [11]. Phytosterols are structurally similar to cholesterol and can inhibit cholesterol absorption. Many randomized controlled trials (RCTs) have confirmed that phytosterols added into fat-based foods can significantly reduce serum total cholesterol (TC) and LDL cholesterol (LDLc) [12–14]. A recent meta-analysis including 84 RCTs suggests that 2.15 g/d phytosterols added in fat matrices lowers LDLc concentration by 8.8% [15]. However, daily intake of capsules containing 2 g phytosterols did not reduce total- or LDL-cholesterol significantly [16], this study emphasized the importance of phytosterols from daily diets in cholesterol lowering effect.

The daily intakes of total phytosterols usually range from 200 to 400 mg in general population [17, 18] and higher daily intake of 500–1000 mg can be reached by vegetarians [19, 20]. However, in most RCTs, the health benefits are observed at a high dose of phytosterols intake (around 2 g/d), which cannot be achieved in habitual diets. In total, there are limited evidence limited evidence on the association of natural dietary intake of phytosterols and anthropometric parameters, blood glucose and serum lipid profiles. This study aims to examine the hypothesis that higher dietary phytosterols improves body weight and metabolic profile in healthy adults.

## Methods

### Internet-based FFQ

In large population, food frequency questionnaires (FFQs) were universally used by researchers [21]. However, impossibility of conducting traditional face-to-face FFQs during a short period make it inconvenient in researches due to time-consuming [22]. Therefore, a portable tool named Internet-based dietary questionnaire for Chinese (IDQC) has been designed and validated at Harbin Medical University by experts of nutrition, epidemiology and bio-statistics [23, 24]. In IDQC, commonly eaten foods were divided into 16 categories (i.e., grains, potatoes, legumes, vegetables, fungus, fruits, seeds and nuts, livestock, poultry, dairy, eggs, fish, snacks, sugar, condiments, and beverages). Reference images of each food item’s weight/volume were exhibited as references to assist the participants in making accurate estimation of food portions. Each participant had to choose the frequency and amount of each subtype of food groups. Finally, the questionnaires were uploaded to a secure website, which is free of access at http://www.yyjy365.org/diet.

### Study population

To restrict the cost of the biochemical measurements and the length of the study, we randomly selected 912 participants from the database of previously published literature [25] (Additional file 1: Figure S1.). This sample size is sufficient to detect differences of the primary outcome between quartiles, at 95% power and 5% level of significance (0.24 mmol/l in TC and 0.24 mmol/l in LDLc). Online questionnaires including age, gender, occupation, education background, income, physical exercise, smoking, drinking and dietary habits during the past 6 months were completed by all participants as previously described [25]. Daily nutrients intakes were then calculated by using the computerized China food composition tables of 2009 [26].

In this study, the following exclusion criteria were applied: (1) missing data of dietary intake; (2) reported extremely low or high energy intake (i.e. < 800 kcal (3349 kJ) or > 5000 kcal (20,934 kJ) for males; < 600 kcal (2512 kJ) or > 4000 kcal (16,747 kJ) for females); (3) Diabetes diagnosis in routine physical examination; (4) Being on a diet in the past 6 months. The study complied with the Declaration of Helsinki and was approved by the Human Research Ethics Committee of the Harbin Medical University (approval code [2015] 006). Online informed consent was obtained from all participants.

### Estimation of phytosterols intake

On the basis of dietary habits of Chinese residents, more than 160 types of food items (including vegetable oils, cereal and its products, beans and its products, vegetables, fruits, nuts and potatoes) in the Chinese food composition table were chosen for measurement. The contents of β-sitosterol, campesterol, stigmasterol, β-sitostanol and campestanol in food were analyzed at Institute of Nutrition and Food Safety, Chinese Centre for Disease Control and Prevention, Beijing, China, using gas chromatograph methods by Han et al. [27, 28]. The database of phytosterols concentrations were established and were linked to the questionnaire data, intake of phytosterols were then estimated using the IDQC. A supplementary table outlining the phytosterols contents of typical foods in Chinese was provided (Additional file 2: Table S1).

### Anthropometric measurements, cut-off points of overweight/obesity and definition of abdominal obesity

Height, bodyweight and waist circumference (WC) were measured by well-trained medical students. Body mass index (BMI) was calculated as body weight in kilograms divided by the square of the height in meters (kg/m^2^). Participants were asked to wear light, thin clothing and no shoes. Body weight and height were measured to the nearest 0.1 kg and 0.1 cm. WC was measured midway between the lowest rib and the iliac crest with a flexible anthropometric tape on the horizontal plane with the participant in standing position, to the nearest of 0.1 cm. After a 10 min rest in a sitting position, systolic blood pressure (SBP) and diastolic blood pressure (DBP) of participants were gauged using a standard mercury sphygmomanometer by nurses of the health examination center, mean values of three times were recorded. The BMI cut-off points in Chinese subjects for overweight (24 ≤ BMI < 28 kg/m^2^) and obesity (BMI ≥ 28 kg/m^2^) were used in our study to define these states [29]. According to the 2006 Guidelines on Preservation and Control Overweight and Obesity in Chinese Adults classification [30], abdominal obesity was defined as WC ≥ 85 cm in men and WC ≥ 80 cm in women.

### Serum collection and laboratory measurements

Fasting blood samples were collected between 8:00 and 9:00 a.m. after fasting overnight. Then, each participant was asked to take 75 g glucose and postprandial blood samples were drawn 2 h later. FBG and 2 h-PG were measured with a hand-held glucometer (ACCU-CHEK, Roche, Shanghai, China). Blood samples were centrifuged at 3000 rpm for 15 min and the serum was used for laboratory measurements immediately. Serum triglyceride (TG), total cholesterol (TC), high density lipoprotein cholesterol (HDLc) and low density lipoprotein cholesterol (LDLc) were determined using a Roche Modular P800 Automatic Biochemical Analyzer (Roche Diagnostics, Mannheim, Germany). Coefficients of variations of lab measurements were presented in supplemental materials.

### Statistical analysis

For descriptive statistics, means and SDs (or frequencies and percentages) were calculated across quartiles of dietary phytosterols and compared using one-way analysis of variance or chi-square test, as appropriate. Bivariate correlation analysis (without variants adjusted) and partial correlation analysis (with adjustment for energy intake) was performed to assess the association of dietary phytosterols and BMI, WC, SBP, DBP, FBG, 2 h-PG, serum TG, TC, HDLc and LDLc. To estimate the odds ratio (OR) and 95% confidence interval (CI) of overweight/obesity and abdominal obesity between quartiles of dietary phytosterols, logistic regression model was used. Age, education, income, labor, exercise status, smoking habits, drinking habits, dietary carbohydrate, fat, protein, cholesterol and fiber intake were adjusted in three models. The statistical analyses were carried out using SAS software (version 9.1; SAS Institute, Cary, NC, USA) and *P* < 0.05 was considered statistically significant.

## Results

### Characteristics and dietary intakes of the study population

The characteristics and dietary intakes of participants were summarized in Table 1. Participants of the 3rd quartile of dietary phytosterols were youngest. Participants of the 4th quartile of dietary phytosterols had a higher percentage of low income and low education level versus the first quartile. For dietary intakes, energy, protein, fat, fiber and cholesterol intake increased through quartiles of dietary phytosterols. Finally, among individuals with higher intake of phytosterols, lower prevalence of overweight/obesity and abdominal obesity were observed.

**Table 1:** Characteristics and dietary nutrients intake of participants by quartiles (Q) of total phytosterols intake

	Quartiles based on total phytosterols intake				
Characteristics	Q1	Q2	Q3	Q4	*P*
Total, n	228	228	228	228	
Age, y	32.4 ± 12.2a	33.5 ± 13.1a	29.4 ± 11.4b	33.2 ± 12.6a	0.002
Gender, M/W	109/119	88/140	106/122	106/122	0.179
Income per month					0.027
< 2000 yuan, n (%)	133 (58.3)	133 (58.3)	154 (67.5)	158 (69.3)	
2000–5000 yuan, n (%)	86 (37.7)	92 (40.4)	70 (30.7)	64 (28.1)	
≥ 5000 yuan, n (%)	9 (3.9)	3 (1.3)	4 (1.8)	6 (2.6)	
Education					< 0.001
Under college, n (%)	46 (20.2)	52 (22.8)	54 (23.7)	72 (31.6)	
Bachelor, n (%)	162 (71.1)	166 (72.8)	170 (74.5)	152 (66.7)	
Master or doctor, n (%)	20 (8.8)	10 (4.4)	4 (1.8)	4 (1.8)	
PAL					0.145
Light, n (%)	58 (25.4)	58 (25.4)	58 (25.4)	70 (32.5)	
Medium, n (%)	160 (70.2)	161 (70.6)	168 (73.7)	154 (67.5)	
Heavy, n (%)	10 (4.4)	9 (3.9)	2 (0.9)	4 (1.8)	
Exercise					0.949
< 10 h/week, n (%)	101 (44.3)	94 (41.2)	88 (38.6)	92 (40.4)	
10–20 h/week, n (%)	98 (43.0)	104 (45.6)	108 (47.4)	106 (46.5)	
≥ 20 h/week, n (%)	29 (12.7)	30 (13.2)	32 (14.0)	30 (13.2)	
Smoking^a^					0.072
Non-smoker, n (%)	206 (90.4)	207 (90.8)	210 (92.1)	194 (85.1)	
Current smoker, n (%)	22 (9.6)	21 (9.2)	18 (7.9)	34 (14.9)	
Drinking^a^					0.636
Non-drinker, n (%)	182 (79.8)	184 (80.7)	184 (80.7)	192 (84.2)	
Current drinker, n (%)	46 (20.2)	44 (19.3)	44 (19.3)	36 (15.8)	
Overweight/obesity, n (%)	100 (43.9)	90 (39.5)	82 (36.0)	74 (32.5)	0.075
Abdominal obesity, n (%)	77 (33.8)	62 (27.2)	59 (25.9)	54 (23.7)	0.092
Dietary intakes					
Energy, kcal	2179.3 ± 528.7a	2271.9 ± 551.5b	2368.9 ± 548.6c	2498.2 ± 494.1d	< 0.001
Protein, g/d	76.1 ± 21.2a	82.9 ± 23.9b	86.9 ± 24.9b	95.2 ± 22.1c	< 0.001
Fat, g/d	60.2 ± 23.0a	69.5 ± 23.7b	74.3 ± 24.7c	86.4 ± 21.7d	< 0.001
Carbohydrate, g/d	344.9 ± 93.6	343.6 ± 89.9	353.0 ± 91.7	355.8 ± 89.8	0.399
Fiber, g/d	14.4 ± 3.9a	17.3 ± 4.4b	19.5 ± 4.9c	23.1 ± 5.2d	< 0.001
Cholesterol, mg/d	382.9 ± 230.5a	461.1 ± 266.9b	445.8 ± 275.0b	504.2 ± 260.4c	< 0.001
Total plant sterols, mg/d	161.9 ± 23.0	231.3 ± 20.1	294.0 ± 18.8	383.0 ± 54.4	< 0.001
β-sitosterol, mg/d	96.4 ± 16.8a	139.9 ± 22.2b	187.9 ± 27.9c	255.7 ± 47.3d	< 0.001
campesterol, mg/d	32.5 ± 13.4a	48.8 ± 20.4b	51.4 ± 26.9b	57.9 ± 29.2c	< 0.001
stigmasterol, mg/d	12.5 ± 5.4a	15.0 ± 5.9b	17.8 ± 7.5c	19.0 ± 7.3d	< 0.001
β-sitostanol, mg/d	9.2 ± 4.4a	13.3 ± 5.6b	18.5 ± 6.6c	27.2 ± 7.5d	< 0.001
campestanol, mg/d	11.3 ± 3.3a	14.4 ± 4.0b	18.3 ± 4.2c	23.2 ± 4.8d	< 0.001

*P* values are for differences across quartiles of total plant sterols intake; Data are mean ± SD or frequency (percentage), a, b, c, d, mean values with unlike letters were significantly different (*P* < 0.05)

Abbreviations: *PAL* physical activity level

^a^ There is no report of quitting smoking or quitting drinking in our population

### Anthropometric parameters, blood glucose and serum lipid profiles of participants

BMI, WC, blood glucose and serum lipid profiles of participants among quartiles of dietary phytosterols were showed in Table 2. Compared with those in the first quartile of dietary phytosterols, participants of the 4th quartile had lower BMI, WC, SBP, DBP, TC and LDLc (*P* < 0.01 for BMI and TC, *P* < 0.05 for WC, SBP, DBP and LDLc). After stratified by gender, similar results were observed in men and women (all *P* < 0.05). There is no significant difference of FBG, 2 h-PG, TG and HDLc among quartiles of dietary phytosterols.

**Table 2:** Anthropometric parameters, blood glucose and serum lipid profiles of participants by quartiles (Q) of total phytosterols intake

	Quartiles based on total phytosterols intake				
	Q1	Q2	Q3	Q4	*P*
Overall, n	228	228	228	228	
BMI, kg/m^2^	23.85 ± 3.17	23.53 ± 3.44	23.05 ± 3.71^*^	22.67 ± 2.76^**^	0.001
WC, cm	80.31 ± 10.21	78.76 ± 8.74	78.45 ± 9.67^*^	78.12 ± 7.91^*^	0.044
SBP, mm Hg	119.51 ± 11.16	117.13 ± 12.42	116.45 ± 11.33	115.04 ± 10.14^*^	0.034
DBP, mm Hg	78.74 ± 8.92	77.32 ± 8.41	76.72 ± 8.93	76.27 ± 7.22^*^	0.046
FBG, mmol/l	4.71 ± 0.81	4.68 ± 0.73	4.59 ± 0.82	4.62 ± 0.78	0.282
2 h-PG, mmol/l	6.42 ± 0.97	6.35 ± 0.81	6.33 ± 0.87	6.27 ± 0.96	0.361
TG, mmol/l	1.68 ± 1.24	1.73 ± 1.31	1.71 ± 1.28	1.75 ± 1.38	0.916
TC, mmol/l	5.25 ± 0.87	5.17 ± 0.78	5.09 ± 0.86	5.01 ± 0.90^**^	0.004
HDLc, mmol/l	1.26 ± 0.33	1.25 ± 0.34	1.29 ± 0.29	1.25 ± 0.36	0.289
LDLc, mmol/l	2.55 ± 0.83	2.42 ± 0.86	2.39 ± 0.79	2.31 ± 0.90^*^	0.042
Men, n	102	102	102	103	
BMI, kg/m^2^	23.91 ± 3.09	23.77 ± 3.25	23.41 ± 3.36	22.75 ± 2.68^*^	0.016
WC, cm	83.45 ± 9.26	81.19 ± 9.62	79.63 ± 9.18^*^	78.58 ± 7.26^*^	0.024
SBP, mm Hg	120.12 ± 12.01	118.03 ± 11.45	117.17 ± 11.45	115.03 ± 9.98^*^	0.047
DBP, mm Hg	79.11 ± 7.92	78.23 ± 8.63	77.87 ± 8.95	77.03 ± 7.15^*^	0.045
FBG, mmol/l	4.75 ± 0.86	4.81 ± 0.78	4.79 ± 0.81	4.72 ± 0.91	0.442
2 h-PG, mmol/l	6.51 ± 0.92	6.66 ± 0.98	6.35 ± 0.78	6.24 ± 0.86	0.296
TG, mmol/l	1.79 ± 1.14	1.73 ± 1.21	1.78 ± 1.24	1.76 ± 1.41	0.855
TC, mmol/l	5.17 ± 0.91	5.06 ± 0.89	5.01 ± 0.81	4.95 ± 0.88^*^	0.028
HDLc, mmol/l	1.27 ± 0.35	1.26 ± 0.38	1.24 ± 0.32	1.23 ± 0.37	0.354
LDLc, mmol/l	2.44 ± 0.86	2.31 ± 0.91	2.25 ± 0.77	2.19 ± 0.86^*^	0.034
Women, n	125	126	126	126	
BMI, kg/m^2^	23.72 ± 3.24	23.41 ± 3.31	22.78 ± 3.74^*^	22.45 ± 2.66^*^	0.021
WC, cm	76.02 ± 8.17	74.28 ± 8.21	73.20 ± 9.12^*^	72.89 ± 7.31^*^	0.047
SBP, mm Hg	118.23 ± 10.93	116.34 ± 12.78	114.96 ± 11.61	113.82 ± 10.21^*^	0.038
DBP, mm Hg	77.71 ± 8.86	76.25 ± 8.52	75.12 ± 8.61	74.78 ± 7.31^*^	0.032
FBG, mmol/l	4.68 ± 0.82	4.59 ± 0.71	4.41 ± 0.78	4.44 ± 0.98	0.341
2 h-PG, mmol/l	6.31 ± 0.91	6.26 ± 0.95	6.31 ± 0.82	6.33 ± 0.91	0.458
TG, mmol/l	1.58 ± 1.31	1.72 ± 1.34	1.66 ± 1.22	1.74 ± 1.33	0.545
TC, mmol/l	5.34 ± 0.76	5.29 ± 0.76	5.18 ± 0.92	5.06 ± 0.93^*^	0.013
HDLc, mmol/l	1.24 ± 0.32	1.24 ± 0.31	1.33 ± 0.29	1.27 ± 0.36	0.128
LDLc, mmol/l	2.67 ± 0.81	2.52 ± 0.83	2.46 ± 0.81	2.41 ± 0.85^*^	0.044

Abbreviations: *BMI* body mass index, *WC* waist circumference, *SBP* systolic blood pressure, *DBP* diastolic blood pressure, *FBG* fasting blood glucose, *2 h-PG* 2 h-postprandial glucose, *TG* triglyceridel, *TC* total cholesterol, *HDLc* high destiny lipoprotein cholesterol, *LDLc* low destiny lipoprotein cholesterol

* *P* < 0.05; ** *P* < 0.01 compared with the 1st quartile

### Correlations between dietary phytosterols and anthropometric parameters, blood glucose and serum lipid profiles

As shown in Table 3, we found negative correlations between total phytosterols and BMI, WC, SBP, DBP, TC and LDLc (all *P* < 0.05). The associations were stronger with adjustment of total energy intake. After stratified by gender, similar trends were observed in male and female (all *P* < 0.05). However, no significant associations between dietary phytosterols and FBG, 2 h-PG, TG and HDLc were observed.

**Table 3:** Correlation analysis of dietary total phytosterols versus anthropometric parameters, blood glucose and serum lipid profiles

	Total phytosterols intake, mg/d			
	Unadjusted		Adjusted^a^	
	*r*	*P*	*r*	*P*
Overall				
BMI, kg/m^2^	−0.181	< 0.001	− 0.226	< 0.001
WC, cm	− 0.106	0.001	− 0.167	< 0.001
SBP, mm Hg	− 0.112	< 0.001	− 0.132	< 0.001
DBP, mm Hg	− 0.109	0.002	−0.134	< 0.001
FBG, mmol/l	−0.024	NS	−0.033	NS
2 h-PG, mmol/l	−0.019	NS	−0.023	NS
TG, mmol/l	−0.033	NS	−0.041	NS
TC, mmol/l	−0.144	< 0.001	−0.183	< 0.001
HDLc, mmol/l	−0.023	NS	−0.029	NS
LDLc, mmol/l	−0.138	< 0.001	−0.167	< 0.001
Men				
BMI, kg/m^2^	−0.212	< 0.001	−0.243	< 0.001
WC, cm	−0.164	< 0.001	−0.206	< 0.001
SBP, mm Hg	−0.117	0.024	−0.136	0.011
DBP, mm Hg	−0.112	0.031	−0.140	0.023
FBG, mmol/l	−0.028	NS	−0.037	NS
2 h-PG, mmol/l	−0.023	NS	−0.029	NS
TG, mmol/l	−0.034	NS	−0.044	NS
TC, mmol/l	−0.147	0.003	−0.191	< 0.001
HDLc, mmol/l	−0.021	NS	−0.023	NS
LDLc, mmol/l	−0.133	0.006	−0.185	< 0.001
Women				
BMI, kg/m^2^	−0.158	< 0.001	−0.210	< 0.001
WC, cm	−0.087	0.047	−0.122	0.006
SBP, mm Hg	−0.106	0.028	−0.129	0.011
DBP, mm Hg	−0.105	0.042	−0.119	0.033
FBG, mmol/l	−0.019	NS	−0.024	NS
2 h-PG, mmol/l	−0.014	NS	−0.018	NS
TG, mmol/l	−0.029	NS	−0.031	NS
TC, mmol/l	−0.137	0.011	−0.179	< 0.001
HDLc, mmol/l	−0.027	NS	−0.033	NS
LDLc, mmol/l	−0.121	0.021	−0.145	0.006

Abbreviations: *BMI* body mass index, *WC* waist circumference, *SBP* systolic blood pressure, *DBP* diastolic blood pressure, *FBG* fasting blood glucose, *2 h-PG* 2 h-postprandial glucose, *TG* triglyceride, *TC* total cholesterol, *HDLc* high destiny lipoprotein cholesterol, *LDLc* low destiny lipoprotein cholesterol, *NS* no significance

^a^ Adjusting for total energy intake

### Correlations between dietary plant sterols and prevalence of obesity

With adjustment for potential dietary and non-dietary cofounders (age, education, income, PAL, physical exercise, smoking and drinking status, dietary carbohydrate, fat, protein, fiber and cholesterol intake), we found higher dietary total phytosterols intake was linked with lower prevalence of overweight/obesity. Data was illustrated in Table 4, compared with the 1st quartile, the multivariable-adjusted ORs of overweight/obesity of the 4th quartile were 0.440 (0.254, 0.763) in overall population, 0.487 (0.234, 0.918) in men and 0.277 (0.124, 0.619) in women. (All *P* < 0.05).

**Table 4:** Association between total phytosterols intake and prevalence of overweight/obesity

Overall	Q1	Q2	Q3	Q4
Participants, n	228	228	228	228
No. of cases, n	100	90	82	74
Intake, mg/d	161.9	231.3	294	383
Crude	1	0.835 (0.575, 1.212)	0.719 (0.493, 1.047)	0.615 (0.420, 0.900)^*^
Model 1	1	0.746 (0.496, 1.122)	0.762 (0.504, 1.151)	0.496 (0.324, 0.761)^**^
Model 2	1	0.708 (0.462, 1.085)	0.711 (0.445, 1.136)	0.440 (0.254, 0.763)^**^
Men	Q1	Q2	Q3	Q4
Participants, n	102	102	102	103
No. of cases, n	49	44	34	32
Intake, mg/d	157.3	233	381.4	301
Crude	1	0.835 (0.480, 1.451)	0.541 (0.307, 0.952)^*^	0.481 (0.272, 0.850)^*^
Model 1	1	0.752 (0.412, 1.373)	0.552 (0.294, 1.035)	0.509 (0.274, 0.946)^*^
Model 2	1	0.778 (0.403, 1.501)	0.559 (0.244, 1.183)	0.487 (0.234, 0.918)^*^
Women	Q1	Q2	Q3	Q4
Participants, n	125	126	126	126
No. of cases, n	52	51	42	42
Intake, mg/d	165.6	229.3	287.6	384.2
Crude		0.955 (0.577, 1.579)	0.702 (0.420, 1.173)	0.702 (0.420, 1.173)
Model 1		0.927 (0.527, 1.631)	0.790 (0.441, 1.414)	0.501 (0.275, 0.911)^*^
Model 2	1	0.758 (0.416, 1.380)	0.537 (0.275, 1.048)	0.277 (0.124, 0.619)^**^

Model 1: adjusting for age, education, income, PAL, exercise status, smoking and drinking habits

Model 2: Model 1 + dietary carbohydrate, fat, protein, fiber and cholesterol intake were adjusted

* *P* < 0.05; ** *P* < 0.01 compared with the 1st quartile

### Correlations between dietary plant sterols and prevalence of abdominal obesity

In this study, we have observed the reverse relationship between dietary phytosterols and abdominal obesity. As shown in Table 5, compared with the 1st quartile, the multivariable-adjusted ORs of abdominal obesity of the 4th quartile were 0.239 (0.127, 0.450); 0.344 (0.144, 0.819) and 0.321 (0.140, 0.571) in overall population, men and women, respectively (All *P* < 0.05). Potential dietary and non-dietary cofounders (age, education, income, PAL, physical exercise, smoking and drinking status, dietary carbohydrate, fat, protein, fiber and cholesterol intake) were rectified.

**Table 5:** Association between total phytosterols intake and prevalence of abdominal obesity

Overall	Q1	Q2	Q3	Q4
Participants, n	228	228	228	228
No. of cases, n	77	62	59	54
Intake, mg/d	161.9	231.3	294	383
Crude	1	0.732 (0.491, 1.093)	0.685 (0.457, 1.025)	0.609 (0.404, 0.918)^*^
Model 1	1	0.806 (0.513, 1.266)	0.566 (0.360, 0.891)^*^	0.490 (0.305, 0.785)^**^
Model 2	1	0.512 (0.302, 0.868)^*^	0.423 (0.259, 0.691)^**^	0.239 (0.127, 0.450)^**^
Men	Q1	Q2	Q3	Q4
Participants, n	102	102	102	103
No. of cases, n	51	43	36	36
Intake, mg/d	157.3	233	381.4	301
Crude	1	0.741 (0.426, 1.289)	0.529 (0.302, 0.927)^*^	0.545 (0.311, 0.957)^*^
Model 1	1	0.657 (0.355, 1.215)	0.520 (0.303, 0.955)^*^	0.510 (0.293, 0.951)^*^
Model 2	1	0.462 (0.233, 0.918)^*^	0.407 (0.191, 0.864)^*^	0.344 (0.144, 0.819)^*^
Women	Q1	Q2	Q3	Q4
Participants, n	125	126	126	126
No. of cases, n	25	23	20	18
Intake, mg/d	165.6	229.3	287.6	384.2
Crude	1	0.893 (0.476, 1.676)	0.755 (0.395, 1.443)	0.667 (0.343, 1.295)
Model 1	1	0.738 (0.354, 1.539)	0.672 (0.305, 1.480)	0.341 (0.151, 0.770)^*^
Model 2	1	0.525 (0.216, 1.276)	0.497 (0.221, 1.118)	0.321 (0.140, 0.571)^**^

Model 1: adjusting for age, education, income, PAL, exercise status, smoking and drinking habits

Model 2: Model 1 + dietary carbohydrate, fat, protein, fiber and cholesterol intake were adjusted

* *P* < 0.05; ** *P* < 0.01 compared with the 1st quartile

## Discussion

To our knowledge, this is the first Internet-based cross-sectional study to investigate associations between dietary phytosterols and anthropometric parameters, blood glucose, serum lipid profiles and prevalence of overweight/obesity and abdominal obesity among healthy subjects. The major finding of this study is that higher dietary phytosterols intake was associated with lower BMI, WC, blood pressure, serum TC and LDLc and lower prevalence of overweight/obesity and abdominal obesity in northern Chinese adults. A moderate dietary intake of total plant sterols was found (267.5 mg/d) in our population. Therefore, this sample can represent the general population.

Lifestyle, including dietary habits and physical exercises, could present a distinct effect on management of bodyweight and lipid/lipoprotein. Here, we generally found a dose-dependent inverse association between phytosterols intake from natural diet and BMI, WC, blood pressure, TC and LDLc in northern Chinese adults. Our findings suggest that higher intake of phytosterols in natural diet may be favorable in the prevention of overweight/obesity and improve cholesterol metabolism. Therefore, reasonable advice of consuming more phytosterol-rich foods may be useful for these patients.

It has been established that high dose of phytosterols can significantly reduce blood cholesterol in RCTs [31]. However, it was still undecided whether low doses of phytosterols from natural diets were effective in management of serum cholesterol. In our reserch, we found that higher dietary phytosterols were related with lower serum TC and LDLc after adjustment for total energy intake. Similar results were observed in two large cross-sectional studies. In the European Prospective Investigation into Cancer and Nutrition (EPIC-Norfolk) study, a 289 mg/d or 281 mg/d (in men/women) increase of phytosterols intake was correlated to 4.1%/2.4% decrease in serum TC and 3.5%/3.0% decrease in LDLc, respectively [32]. Another study including 37,150 men and 40,502 women presents 2.6%/3.5% lower TC and 3.1%/3.2% lower LDLc in highest quintile in comparison with the lowest quintile of plant sterols in men and women, respectively [33].

The underlying mechanism of hypocholesterolemic activity of phytosterols was summarized in a recent review [34]. It is well accepted that phytosterols inhibit the intestinal absorption of dietary cholesterol due to their greater affinity for micelles [35]. Therefore, more cholesterol and its metabolites were excreted by feces. Recent studies also suggest that phytosterols might lower LDLc by upregulating intestinal cholesterol efflux transporters and receptor-mediated lipoprotein cholesterol uptake in response to the reduced supply of exogenous cholesterol [34]. Moreover, in high fat diet feeding hamsters, phytosterols supplementation leads to a remarkable increase in the biliary bile acid:cholesterol ratio, induces the expression of CYP7A1, which governs the synthesis of bile acid and controls its activity at the transcriptional level [36], and normalizes the expression of HNF4A, a key controller of bale acid metabolism [37, 38], indicating that phytosterols enhance bile acid synthesis.

Another important finding of this study was the lower SBP and DBP of the 4th quartile of dietary phytosterols than in the 1st quartile. The lower SBP was most likely interpreted by the lower body weight. The benefits of the decrease in blood pressure are apparent, because lower blood pressure has been shown to lower risk of cardiovascular morbidity [39]. Coker et al. reported that dietary supplementation of essential amino acids and phytosterols may reduce risk for metabolic syndrome and cardiovascular diseases by promoting the reductions of blood lipids and improving insulin resistance in overweight individuals with hypertriglyceridemia [40]. In our work, we did not observe significant correlations in FBG or 2 h-PG because, at least in part, diabetic patients were excluded from the study, and both FBG and 2 h-PG were in normal ranges at baseline.

The major limitation of this study is the cross-sectional and retrospective design which might have limitations in the confirmation of causal relationship. Therefore, long term cohort studies for verification of this issue are needed. Secondly, we included only those subjects who lived in Harbin, in consideration of the large population and vast territory of China, we should be rigorous to extrapolate this result to the general population.

In spite of those defects, this is the first web-based FFQ study that has investigated this issue. In our previous study, there were significantly positive correlations in the dietary intakes of 9 food groups and 23 nutrients between the IDQC and 3-day diet records [24], these data suggested that the intake of phytosterols assessed by using IDQC might reflect a long-term and stable habitual intake of phytosterols from habitual diets in this population. We firstly reported that higher dietary phytosterols were associated with lower BMI, WC, blood pressure, serum TC and LDLc and lower prevalence of overweight/obesity and abdominal obesity, which provided practical significances in nutritional counseling as the importance for prevention of dyslipidemia, obesity and cardiovascular diseases. For overweight/obese and hypercholesterolemia clients, advice of consuming more plant sterols-rich foods may be useful.

## Conclusions

Overall, our study firstly reported that dietary phytosterols were negatively associated with BMI, WC, SBP, DBP, serum TC and LDLc. Higher dietary phytosterols were also associated with lower prevalence of overweight/obesity and abdominal obesity. Further studies are needed to verify the causal association and explore the potential mechanism.

## Additional files

Additional file 1: Flow of the study population. (JPEG 45 kb)

Additional file 2: **Table S1.** The phytosterols contents in commonly consumed foods of China. (DOCX 19 kb)

## Abbreviations

2 h-PG: 2 h-postprandial glucose
BMI: Body mass index
CI: Confidence interval
CVDs: Cardiovascular diseases
DBP: Diastolic blood pressure
FBG: Fasting blood glucose
FFQ: Food frequency questionnaire
HDLc: High density lipoprotein cholesterol
IDQC: Internet-based dietary questionnaire for Chinese
LDLc: Low density lipoprotein cholesterol
OR: Odds ratio
RCTs: Randomized controlled trials
SBP: Systolic blood pressure
TC: Total cholesterol
TG: Triglycerides
WC: Waist circumference
