# Supplementary figures and images for: Associations of dietary phytosterols with blood lipid profiles and prevalence of obesity in Chinese adults, a cross-sectional study
Source: Lipids Health Dis. 2018 Mar 16;17:54. doi: 10.1186/s12944-018-0703-y (PMC5857105; doi:10.1186/s12944-018-0703-y)

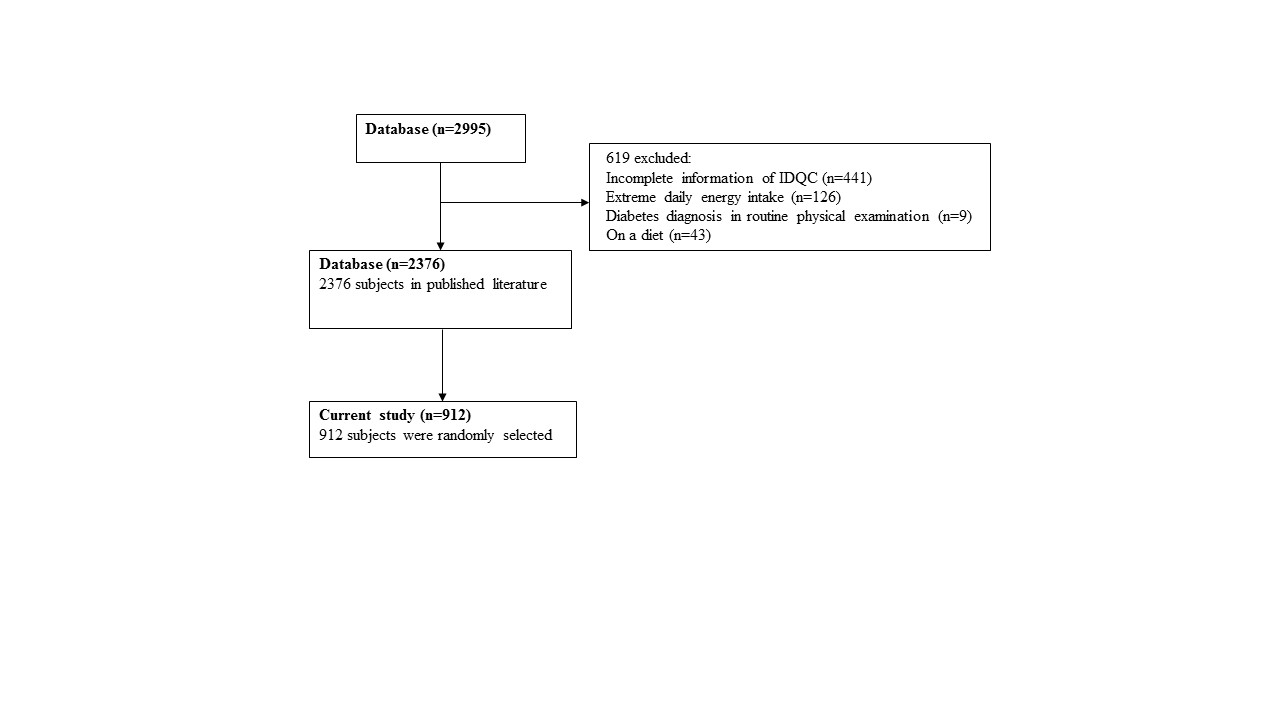

Supplement: Supplementary file 1 — Flow of the study population. (JPEG 45 kb) [file 12944_2018_703_MOESM1_ESM.jpg]
