# Supplementary material for: Associations of dietary phytosterols with blood lipid profiles and prevalence of obesity in Chinese adults, a cross-sectional study
Source: Lipids Health Dis. 2018 Mar 16;17:54. doi: 10.1186/s12944-018-0703-y (PMC5857105; doi:10.1186/s12944-018-0703-y)
Supplement: Supplementary file 2 — Table S1. The phytosterols contents in commonly consumed foods of China. (DOCX 19 kb) [file 12944_2018_703_MOESM2_ESM.docx]

| Table S1 The phytosterols contents in commonly consumed foods of China | | | | | | |
| --- | --- | --- | --- | --- | --- | --- |
| Foods | β-sitosterol | Campesterol | Stigmasterol | β-sitostanol | Campestanol | Total |
| Flour | 34.51 | 8.28 | 1.43 | 10.37 | 5.01 | 59.60 |
| Rice | 6.60 | 2.21 | 1.85 | 2.25 | 0.75 | 13.66 |
| Corn | 22.54 | 5.19 | 2.32 | 23.88 | 6.54 | 60.47 |
| Soybean | 64.98 | 20.95 | 16.30 | 6.50 | 2.16 | 110.89 |
| Carrot | 13.97 | 1.94 | 2.99 | 0.23 | 0.16 | 19.29 |
| Cabbage | 11.61 | 2.01 | 10.37 | - | - | 23.99 |
| Tomato | 2.94 | 0.62 | 1.88 | 0.73 | - | 6.17 |
| Spinach | 5.37 | 0.78 | 2.90 | 0.76 | 0.71 | 10.52 |
| Apple | 10.18 | 0.43 | 0.18 | 0.10 | - | 10.89 |
| Orange | 23.33 | 3.17 | 1.45 | 0.52 | - | 28.47 |
| Pear | 13.06 | 0.24 | 0.27 | 0.37 | - | 13.94 |
| Potato | 2.11 | 0.12 | 0.72 | 0.63 | - | 3.58 |
| Peanut | 111.44 | 26.10 | 12.71 | 17.13 | 2.55 | 169.93 |
| Sunflower seed | 199.15 | 33.05 | 19.45 | 22.61 | 1.89 | 276.15 |
| Filbert | 153.90 | 7.45 | 2.53 | 10.00 | - | 173.88 |
| Soybean oil | 175.60 | 58.05 | 56.10 | 16.08 | 1.52 | 307.35 |
| Peanut oil | 164.73 | 35.60 | 23.00 | 21.79 | - | 245.12 |
| Olive oil | 216.36 | 10.58 | 3.20 | - | - | 230.14 |

In this study, more than 160 types of food items (including vegetable oils, cereal and its products, beans and its products, vegetables, fruits, nuts and potatoes) in the Chinese food composition table were chosen for measurement.

**CV of lab measurements**

For the biochemical analyses, the intra- and inter-assay coefficients of variations (CV%) of different parameters were listed as follows: serum glucose (intra-day CV%, 4.38%; inter-day CV%, 6.85%); TC (intra-day CV%, 4.16%; inter-day CV%, 6.27%); TG (intra-day CV%, 4.94%; inter-day CV%, 7.03%); HDLc (intra-day CV%, 4.85%; inter-day CV%, 6.85%); LDLc (intra-day CV%, 4.53%; inter-day CV%, 6.16%).
